# A modified Tessari method for producing more foam

**DOI:** 10.1186/s40064-016-1769-5

**Published:** 2016-02-19

**Authors:** Jie Xu, Yi-fei Wang, An-wei Chen, Tao Wang, Shao-hua Liu

**Affiliations:** School of Stomatology, Shandong University, No. 44, Wenhuaxi-Road, Jinan, 250012 People’s Republic of China; Department of Oral and Maxillofacial Surgery, Stomatology Hospital of Jinan, No. 101, Jingliu-Road, Jinan, 250012 People’s Republic of China; Department of Oral and Maxillofacial Surgery and Institute of Dental Medicine, Qilu Hospital, Shandong University, No. 107, Wenhuaxi-Road, Jinan, 250012 People’s Republic of China; Department and Institute of Dental Medicine, Qilu Hospital, Shandong University, Wenhuaxi-Road, Jinan, 250012 People’s Republic of China

**Keywords:** Sclerosing foam, Tessari technique, Modified Tessari method, Sclerotherapy, Venous malformations

## Abstract

This study aimed to develop a modified Tessari method for producing more sclerosing foam in treatment of extensive venous malformations. Sclerosing foam was produced by using Tessari method and the modified Tessari method. The procedure of the later was as follows: prepared foam in a sclerosant–air ratio of 1:4; connected three disposable 10 ml syringes to two medical three-way taps; drawn 4 ml of liquid sclerosant into one syringe and 16 ml averagely of air into the other two; then moved the plungers of all syringes back and forth for 20 times to produce sclerosing foam. The volume and foam half time (FHT) of foam produced by the two methods were compared. The average volume of sclerosing foam produced by Tessari method and the modified Tessari method were 9.8 and 19.7 ml, and assessed to have statistical difference. The FHT of foam produced by the two methods were 120 and 150 s, and assessed to have statistical difference. In conclusion, the modified Tessari method could produce more fresh and stable sclerosing foam.

## Background

During the treatment of venous malformations (VMs) using foam sclerotherapy, one of the most popular methods to produce foam was the Tessari method which used pumping cycles of liquid and air in-and-out of a double syringe system (Nastasa et al. 2015; Wolmann 2010; Tessari 2001, 2005). In clinical treatment, sclerosing foam was prepared usually by using two 10 ml syringes; however, no more than 10 ml of sclerosing foam could be produced at a time in this way. Tessari method was originally used in the treatment of varicose veins, in most cases of which 10 ml of fresh sclerosing foam might be sufficient (Tessari et al. 2001). However, this volume might be insufficient in treating some VMs, especially when the region of lesion was extensive. Sufficient fresh sclerosing foam was needed for the treatment of extensive VMs.

There were several ways to get more fresh sclerosing foam, for example, producing foam once more, using bigger syringes or more operators and devices to produce simultaneously. However, all these methods had more or less defects. Here, we reported a modified Tessari method by using three 10 ml syringes which could produce more fresh foam at a time; the volume and the stability of foam produced by the two methods were compared. Foam half time (FHT), the time when half of the original liquid sclerosant was reverted to liquid state, was adopted to assess the stability of the foam.

## Methods

Disposable 10 ml syringes, medical three-way taps and 1 % lauromacrogol injection (Shanxi Tianyu Pharmaceutical Co., Ltd.) were used in this study.

**Fig. 1:**
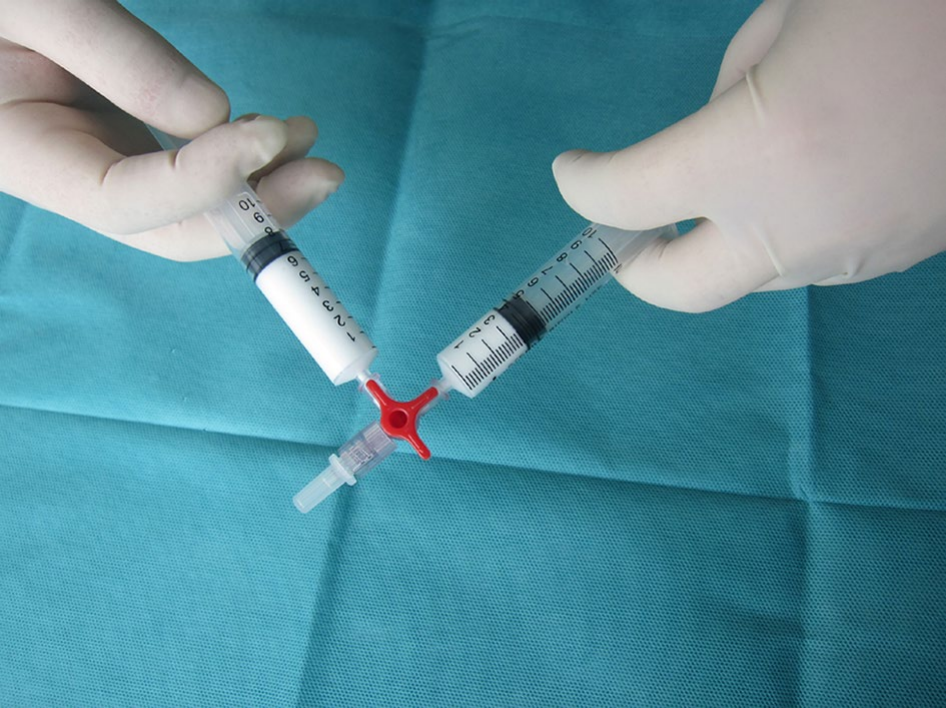
Tessari method

Procedure of Tessari method (Tessari et al. 2001): drawn 2 ml of liquid sclerosant into one 10 ml syringe and 8 ml of air into the other 10 ml syringe; connected both syringes to a medical three-way tap; then, moved the plungers of both syringes back and forth for 20 times to produce sclerosing foam (Fig. 1).

**Fig. 2:**
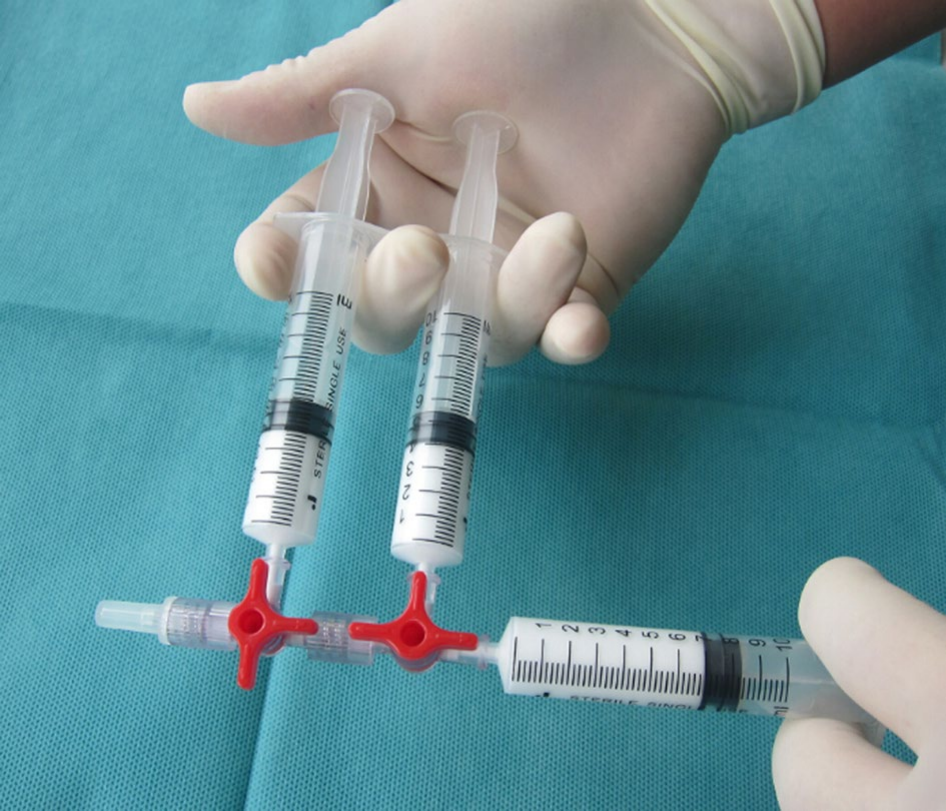
The modified Tessari method

Procedure of the modified Tessari method: drawn 4 ml liquid sclerosant into a 10 ml syringe and 16 ml air averagely into the other two 10 ml syringes; then connected all syringes to two parallel medical three-way taps; then moved the plungers of all syringes back and forth for 20 times to produce sclerosing foam (Fig. 2).

Detection of the foam volume and the foam FHT: Once the foam was produced, it was immediately pushed into 10 and 20 ml scalar syringes respectively, the scalar syringes were placed vertically with the piston beneath. Macroscopic appearance of the foam was observed and volume of the foam was recorded. The foam in the syringes gradually separated into liquid and air, with the liquid sclerosant gathered at the bottom, when half volume of the liquid sclerosant was reverted, the time as FHT was recorded.

The detection above was measured respectively for 10 times by the same operator. Each foam was prepared by using new sclerosant and all experiments were performed at an ambient temperature of 25 °C. Experimental values were compared by an independent-samples t test and analysed using SPSS 19.0. A p value ≤0.05 was considered significant.

## Results

Right after production, the bubbles in foam were small and homogenous, when the foam began to re-convert to a liquid and air separated mix, foam bubbles got bigger and inhomogeneous, eventually foam converted to two components: liquid and air.

The average volume of sclerosing foam produced by Tessari method and the modified Tessari method were 9.8 and 19.7 ml, and assessed to have statistical difference.

The FHT of foam produced by Tessari method and the modified Tessari method were 120 and 150 s, and assessed to have statistical difference (Table 1).

**Table 1:** Average volume and foam half time (FHT) of foam produced by Tessari method and modified Tessari method

	Tessari method	The modified Tessari method
The average volume (ml)^a^	9.8 ± 0.2	19.7 ± 0.3
The FHT (s)^a^	120 ± 4.13	156 ± 6.85

^a^The average volume and FHT were expressed as mean values ± standard deviation

## Discussion

Owing to its high efficiency, safety and economical advantages, foam sclerotherapy was widely used in treatment of vascular abnormalities (Luebke and Brunkwall 2008; Myers and Roberts 2009; Park et al. 2014; Van der Vleuten et al. 2014; Wolmann 2010). The high efficiency of foam sclerotherapy was closely related to the physical obstruction of blood flow and the displacing the intravascular blood, which extended extent of contaction between the sclerosants and the vessel wall (Ikponmwosa et al. 2010; Wolmann 2010; Eckmann 2009; Van Deurzen et al. 2011). Therefore, the therapeutic effects related to whether the cavity of VMs could be replete by fresh sclerosing foam; In our clinical work, sometime more than 10 ml fresh sclerosing foam was needed for extensive lesion, some authors found that 10 ml or more sclerosing foam was safety and efficacy, and no serious complications were observed (Stimpson et al. 2012; Chen et al. 2015). In our experience, the most common complications were temporal pain with injection, swelling after injection and skin ulcer in some patients.

In our experimental and clinical experience, it was inconvenient to use 20 ml syringes to produce foam; and other authors had reported that 10 ml syringes were the best choice to produce foam considering normal handling and foam stability (Wolmann 2010; Nastasa et al. 2015). Tessari method using two 10 ml syringes had become one of the most popular ways to produce foam. In clinical treatment, no more than 10 ml of foam could be produced at a time by using two 10 ml syringes, which might be insufficient in treating some extensive VMs.

To get more foam at a time, we might repeat the producing procedure once more or add more operators and devices. But the process of injecting foam into extensive VMs might be interrupted. During the interval between two adjacent injections, the previous foam would be washed away by the blood. Therefore, injecting sufficient foam at a time into the cavity of extensive VMs produced better therapeutic effects (Van Deurzen et al. 2011). Furthermore, there was another disadvantage when using the same syringe to prepare foam for the second time, it would be hard for the plunger to slide smoothly during preparation and injection. Adding more operators and devices to produce foam simultaneously meant to use more human and material resources. The modified Tessari method could produce about 20 ml fresh foam at a time which, in our clinical experience, would be sufficient in treating some of extensive VMs.

FHT was a key and usually used parameter of the stability of sclerosing foam. In this study, the FHT of sclerosing foam produced by the modified Tessari method was longer than that by Tessari method, showing that the sclerosing foam produced by the modified Tessari method was more stable.

## Conclusions

The modified Tessari method could produce more fresh and stable sclerosing foam at a time which was profitable in treating extensive VMs.

## Abbreviations

VMs: venous malformations
FHT: foam half time
